# Establishing the cognitive signature of human brain networks derived from structural and functional connectivity

**DOI:** 10.1007/s00429-018-1734-x

**Published:** 2018-08-17

**Authors:** JeYoung Jung, Maya Visser, Richard J. Binney, Matthew A. Lambon Ralph

**Affiliations:** 1 Neuroscience and Aphasia Research Unit (NARU), Division of Neuroscience and Experimental Psychology, School of Biological Sciences (Zochonis Building), University of Manchester, Brunswick Street, Manchester, M13 9PL UK; 2 Grupo de Neuropslcología y NeuroLmagen Functional, University Jaume I, Castellón de la Plana, Castellón Spain; 3 School of Psychology, Bangor University, Bangor, UK

**Keywords:** Associative cortex, Higher cognitive function, Structural connectivity, Functional connectivity, Representational similarity analysis

## Abstract

Numerous neuroimaging studies have identified various brain networks using task-free analyses. While these networks undoubtedly support higher cognition, their precise functional characteristics are rarely probed directly. The frontal, temporal, and parietal lobes contain the majority of the tertiary association cortex, which are key substrates for higher cognition including executive function, language, memory, and attention. Accordingly, we established the cognitive signature of a set of contrastive brain networks on the main tertiary association cortices, identified in two task-independent datasets. Using graph-theory analysis, we revealed multiple networks across the frontal, temporal, and parietal cortex, derived from structural and functional connectivity. The patterns of network activity were then investigated using three task-active fMRI datasets to generate the functional profiles of the identified networks. We employed representational dissimilarity analysis on these functional data to quantify and compare the representational characteristics of the networks. Our results demonstrated that the topology of the task-independent networks was strongly associated with the patterns of network activity in the task-active fMRI. Our findings establish a direct relationship between the brain networks identified from task-free datasets and higher cognitive functions including cognitive control, language, memory, visuospatial function, and perception. Not only does this study support the widely held view that higher cognitive functions are supported by widespread, distributed cortical networks, but also it elucidates a methodological approach for formally establishing their relationship.

**Electronic supplementary material:**

The online version of this article (10.1007/s00429-018-1734-x) contains supplementary material, which is available to authorized users.

## Introduction

Contemporary neuroimaging has shown that human cognition is supported by widespread, distributed cortical networks. Examining neural function as a network provides new insights about large-scale communication in the human brain. It establishes a foothold to investigate how brain connectivity is related to human behavior and how this organization can be influenced by neurodevelopment, neurological disorders, and aging (Biswal et al. 1995; Bassett and Bullmore 2009; Bullmore and Sporns 2009; Menon 2013; Fair et al. 2007). As reviewed below, an ever-growing range of methods are now available for deriving network structure from MRI data. There is, however, a critical missing component: there is still a need to evaluate the derived networks’ cognitive signatures formally if the link between brain networks and higher cognition is to be truly established. This was the goal of the present study.

Human higher cognition arises from coordinated action between a widespread, distributed neural network within the main tertiary association cortices including the frontal, temporal, and parietal lobes. For example, executive function is supported by the subset of frontal and parietal regions (Seeley et al. 2007; Duncan 2010) and language functions arise from an extensive brain system including Broca’s and Wernicke’s areas, as well as other prefrontal, temporal, and parietal regions (Binder et al. 1997; Friederici 2011). Memory system is also embedded in frontal, parietal, and medial temporal areas (Alvarez and Squire 1994). Thus, we examined cognitive signatures derived from structural and functional connectivity between the major associative cortices in the current study.

Recent advances in functional magnetic resonance neuroimaging (fMRI) have demonstrated that human functional networks using task-related fMRI and resting-state fMRI (rsfMRI). rsfMRI methods have been a popular way to define intrinsic networks by examining the pattern of coactivation between the functional time-series of anatomically remote brain regions (Biswal et al. 1995; Damoiseaux et al. 2006; Fox and Raichle 2007). Many rsfMRI studies have reported and replicated the identification of functionally linked networks during rest (resting-state networks) including the primary visual network, auditory network, motor network, and higher order cognitive networks using model-dependent methods (seed-based analysis) and model-free methods (independent component analysis; ICA) (Biswal et al. 1995; Damoiseaux et al. 2006; Beckmann et al. 2005; Fox and Raichle 2007). There are, however, a number of remaining challenges. Some of these methods are subjective in nature, while the outputs of data-driven approaches (e.g., ICA components) can be more difficult to understand than the seed-based analyses as they can contain complex representations of the data (Fox and Raichle 2007). Furthermore, studies of rsfMRI-defined networks often visually compare the outcomes to task-based cognitive fMRI studies but rarely formally test the true cognitive functions of the rsfMRI networks. Recently, studies have compared rsfMRI networks with task-evoked networks directly; some investigations showed high correspondence between them (Cole et al. 2014; Smith et al. 2009) and the others not (Buckner et al. 2013; Bolt et al. 2017). Here, we applied a new method for evaluating the functional characteristics—“cognitive signature” for each of the identified interconnected networks, focusing on the associative cortices. We use the term cognitive signature to mean the functional profile or fingerprint of each brain network with respect to which collection of cognitive activities they are engaged in.

A second, related aim of the current study was the relationship between structural (white matter) and functional (correlated fMRI time-series) connectivity. A number of studies have demonstrated a direct association between functional and structural connectivity in the human brain by combining rsfMRI and diffusion neuroimaging (for a review, see Deco et al. 2011; Damoiseaux and Greicius 2009). On a whole-brain scale, a recent study reported that resting-state networks were structurally connected by known white matter tracts (van den Heuvel et al. 2009). Moreover, by utilizing graph-theory analysis, it has been demonstrated that brain areas with a higher degree of structural connectivity also showed a higher level of functional connectivity, supporting the proposal that functional connectivity is, at least in part, heavily constrained by the structural connectivity (Hagmann et al. 2007; Honey et al. 2009; Jung et al. 2016). It should be noted, however, that the brain networks tested in these studies are often restricted to the primary sensory networks and/or default mode network (DMN); thus, it is important to extend the exploration to the higher cognitive networks commonly observed in task-active fMRI. To quantify and compare the “cognitive signature” for different datasets, we employed representational similarity analysis (RSA) (Kriegeskorte and Kievit 2013). RSA is pattern information analysis that compares representational geometries computed from different sources of information, including brain regions, stimuli, conceptual and computational models, and behaviors (Kriegeskorte et al. 2008).

Here, we investigated the cognitive signature of higher cognitive networks derived from task-independent data, including both functional (rsfMRI) and structural connectivity (DWI). First, we defined 43 cytoarchitectonically and anatomically defined regions of interest (ROIs) on the lateral associative cortices in the left hemisphere including frontal, temporal, and parietal lobes, given that they are the main associative cortices serving higher cognition such as executive function, language, memory, and attention. Second, we employed probabilistic tractography of distortion-corrected DWI (Embleton et al. 2010) and seed-based analysis of dual-echo rsfMRI (Halai et al. 2014) to overcome the signal dropout and image distortion around the anteroventral temporal areas. Then, task-independent connectivity matrices were constructed from rsfMRI (functional connectivity matrix) and tractography (structural connectivity matrix) data and analyzed using graph-theory analysis. The networks resulting from the graph-theory analysis were tested for their cognitive features with respect to three task-dependent fMRI datasets. Finally, we utilized RSA (Kriegeskorte and Kievit 2013) in a new way to compare quantitatively the activation similarity patterns found in task-dependent fMRIs to the similarity pattern predicted by task-independent networks—thus formally testing the hypothesis that brain structure shapes its functions.

## Materials and methods

### Defining networks

We defined task-independent networks from two datasets: diffusion-weighted imaging (DWI) data and resting-state functional magnetic resonance imaging (rsfMRI) data. Higher cognitive functions such as language, memory, and executive control arise from the associative cortices including the frontal, temporal, and parietal lobes. For example, language functions are embedded in a widely distributed network across the prefrontal, temporal, and parietal cortex (Price 2010; Binder et al. 2009). Until recently, among these areas, the rostral temporal cortices have been disregarded due to the geometric distortion induced by magnetic susceptibility in neuroimaging (Olman et al. 2009; Embleton et al. 2010). The rostral temporal lobe plays an important role in semantic memory, language, and visual processing (Binney et al. 2012; Shimotake et al. 2014). Therefore, in the current study, we utilized datasets that overcome the magnetic susceptibility artifacts by adopting new and advanced imaging techniques, including distortion-corrected diffusion-weighted imaging (DWI) and fMRI (Embleton et al. 2010) as well as dual-echo fMRI (Halai et al. 2014).

#### Task-independent structural connectivity: tractography network

Diffusion-weighted images were acquired in 24 healthy volunteers (11 females; mean age 25.9, range 19–47) without any record of neurological or psychiatric disorders, a dataset described previously and utilized for various tractography-related explorations (Cloutman et al. 2012; Binney et al. 2012; Jung et al. 2016; Bajada et al. 2015, 2017). All participants were right-handed, as assessed by the Edinburgh Handedness Inventory (Oldfield 1971). They gave written informed consent to the study protocol, which had been approved by the local ethics committee of the University of Manchester.

Imaging data were acquired on a 3 T Philips Achieva scanner (Philips Medical System, Best Netherlands), using an eight-channel SENSE head coil. DWI was acquired using a pulsed gradient spin echo-planar sequence, with TE = 59 ms, TR ≈ 11,884 ms, *G* = 62 mTm^−1^, half scan factor = 0.679, 112 × 112 image matrix reconstructed to 128 × 128 using zero padding, reconstructed resolution 1.875 × 1.875 mm, slice thickness 2.1 mm, 60 contiguous slices, 61 non-collinear diffusion sensitization directions at *b* = 1200 smm^−2^ (∆ = 29.8 ms, *δ* = 13.1 ms), 1 at *b* = 0, SENSE acceleration factor = 2.5. Acquisitions were cardiac gated using a peripheral pulse unit positioned over the participants’ index finger or an electrocardiograph. For each gradient direction, two separate volumes were obtained with opposite polarity *k*-space traversal with phase encoding in the left–right/right–left direction to be used in the signal distortion correction procedure (Embleton et al. 2010). A co-localized T2 weighted turbo spin echo scan was acquired with in-plane resolution of 0.94 × 0.94 mm and slice thickness 2.1 mm, as a structural reference scan to provide a qualitative indication of distortion correction accuracy. A high-resolution T1-weighted 3D turbo field echo inversion recovery image (TR ≈ 2000 ms, TE = 3.9 ms, TI = 1150 ms, flip angle 8°, 256 × 205 matrix reconstructed to 256 × 256, reconstructed resolution 0.938 × 0.938 mm, slice thickness 0.9 mm, 160 slices, SENSE factor = 2.5), was obtained for the purpose of high-precision construction of anatomically based ROIs.

**Fig. 1:**
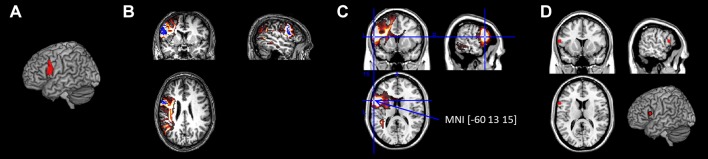
An example of how the region of interest was computed for the tractography and functional connectivity analysis. **a** The BA44 cytoarchitectural map. **b** The transformed BA44 map into a participant’s native space (blue) and its probability map (red-yellow). **c** The group average probability map of BA 44 and a voxel with the highest connectivity value (> 18,000). **d** The BA 44 ROI of functional connectivity analysis from the tractography data

To construct a network across the ventral/lateral prefrontal, temporal, and parietal cortices, we defined 43 regions of interest (ROIs) based on anatomical landmarks and cytoarchitectural maps (Fig. 1a; Fig. S1). Unconstrained probabilistic tractography was performed using the PICo software package (Parker and Alexander 2005), sampling the orientation of probability density functions (PDFs) which was generated using constrained spherical deconvolution (Tournier et al. 2008) and model-based residual bootstrapping (Jeurissen et al. 2011; Haroon et al. 2009). 20,000 Monte Carlo streamlines were initiated from each voxel in each prefrontal, temporal, and parietal ROI. Step size was set to 0.5 mm. Stopping criteria for the streamlines were set so that tracking terminated if pathway curvature over a voxel was greater than 180°, or the streamline reached a physical path limit of 500 mm.

A single whole-brain probabilistic map was generated for each of the 43 ROIs for each participant. Probability maps were masked with each ROI and the maximum connectivity value (ranging from 0 to 20,000) was extracted. Thereby, we obtained a single probability estimate of a pathway between each pair of regions. These values were placed into an individual-specific matrix. The matrix contained two probability estimates for each pair of regions because tracking was performed in both directions (e.g., region A to region B and region B to region A). We combined these two probability estimates to form a single probability estimate for each pair of regions and for each participant. Then, the connectivity matrices were subjected to a double threshold to ensure that only connections with high probability in the majority of participants were considered. For the first-level individual threshold, following the approach described by Cloutman et al. (2012), the *λ*-value of the Poisson distribution identified was used to determine a threshold value at *p* = 0.05. For the second-level group threshold, we used a criteria for consistency (over 50% of participants, i.e., at least 12/24 participants).

The resultant group-level streamline-based connectivity matrix was analyzed using a graph-theory approach (Rubinov and Sporns 2010). The adjacency matrix of ROIs (nodes) and connections (edges) consisted of 43 nodes and 43 × 43 edge binary values. To detect modules within the prefrontal, temporal, and parietal structural connectivity, modularity was computed by the Brain Connectivity Toolbox (Rubinov and Sporns 2010, http://www.brain-connectivity-toolbox.net). Modularity is based on the difference between the number of edges found within modules and the number of edges predicted to lie within modules if all edges in the network were distributed at random. Therefore, this modularity measure quantifies the strength of division of a network into modules.

#### Task-independent functional connectivity: rsfMRI network

Resting-state fMRI images were acquired in 78 healthy volunteers (57 females; mean age 25.2, range 20–44) without any record of neurological or psychiatric disorders. All participants were right-handed, as assessed by the Edinburgh Handedness Inventory (Oldfield 1971). They gave written informed consent to the study protocol, which had been approved by the local ethics committee of the University of Manchester. These data have been reported in recent examination of the semantic network (Jackson et al. 2016).

Imaging data were acquired on a 3 T Philips Achieva scanner (Philips Medical System, Best Netherlands), using a 32 channel SENSE head coil with a sense factor of 2.5. Participants were instructed to keep their eyes open and look at the fixation cross during the scanning. To cover the whole brain without signal dropout around the rostral temporal cortices, a dual-echo fMRI protocol was performed (Halai et al. 2014). This involves parallel acquisition at a short echo (12 ms) leading to less signal loss in areas of high magnetic susceptibility and a standard long echo (35 ms) to maintain high contrast sensitivity throughout the brain. The results from the two echoes were combined using linear summation, previously shown to be optimal (Halai et al. 2014; Poser et al. 2006). The fMRI parameters included 42 slices, 80 × 80 matrix, 240 × 240 × 126 mm FOV, in-plane resolution 3 × 3, slice thickness 4 mm. 130 volumes were collected over 6.25 min. T1-weighted structural images were acquired using a 3D MPRAGE pulse sequence with 200 slices, in-planed resolution 0.94 × 0.94 m slice thickness 1.2 mm, TR = 8.4 ms, TE = 3.9 ms.

Pre-processing was performed using SPM8. The first two volumes were discarded to allow for magnetic saturation effects. The images were slice-time corrected, realigned, and coregistered to the participant’s T1 using SPM8. Censoring was applied using a threshold of greater than 3 mm of translation or 1 degree of rotation, which resulted in the exclusion of 6 participants from further analysis. The images were normalized using DARTEL, smoothed with a 8 mm full-width half maximum (FWHM) Gaussian kernel, and filtered at 0.01–0.08 Hz using Functional Connectivity (CONN) Toolbox (http://web.mit.edu/swg/software.htm). Nuisance covariates were regressed out including 24 motion parameters, white matter, CSF and global tissue signal, and also the performance of linear detrending. The 24 motion parameters were calculated from the 6 original motion parameters using Volterra expansion (Friston et al. 1996) and have been shown to improve motion correction compared to the 6 parameters alone (Yan et al. 2013; Power et al. 2014). Additional covariates were included for outlier time points with *Z*-score greater than 2.5 from the mean global power or more than 1 mm translation as identified using the ARtifact detection Tools software package (ART; http://www.nitrc.org/projects/artifact_detect).

To construct an equivalent network to the tractography network, we defined ROIs using the tractography group results (Fig. 1b). The averaged group-level probabilistic maps for each ROI were thresholded with the connectivity value higher than 18,000 (the maximum connectivity value = 20,000), which resulted in single voxel per ROI (Fig. 1c). Based on the coordinates of each ROI voxel, we selected the nearest gray matter site and defined a 5 mm sphere for each ROI (Fig. 1d). Using the CONN Toolbox (http://web.mit.edu/swg/software.htm), the temporal correlation between BOLD signals among ROIs was computed for each participant. Pre-processed images were registered in the toolbox with 43 ROIs. The functional connectivity analysis provided ROI-to-ROI connectivity estimations. The ROI-to-ROI correlation coefficients were transformed into Fisher’s *Z*-scores and used to construct an association matrix of 43 nodes (ROIs) and 43 × 43 edge values (transformed *Z*-scores) at the individual level. Then we averaged the *Z*-scores across individuals to obtain a group-level matrix. Finally, the averaged *z*-score matrix was converted back to correlation values, which resulted in the group-level association matrix. The obtained association matrix consisted of a set of correlation values ranging from − 1 to 1. The mechanisms of the negative correlation have not been understood yet and several studies demonstrated that the negative correlation could be an artifact caused by a global signal regression procedure (Giove et al. 2009; Murphy et al. 2009; Weissenbacher et al. 2009). Thus, in the current study, we constructed the networks for only positive correlations.

The group-level functional connectivity-based matrix was analyzed using the Brain Connectivity Toolbox (Rubinov and Sporns 2010, http://www.brain-connectivity-toolbox.net) to detect modules within the network. The association matrix of ROIs (nodes) and connections (edges) consisted of 43 nodes and 43 × 43 edges with weighted values. The modularity was assessed by the Rubinnov–Sporns algorithm (Rubinov and Sporns 2011). Because the modularity algorithm is heuristic and produces minimally varied partitions from run to run, 1000 iterations were run to obtain the optimal partitions in the network.

### Verifying task-independent networks

To test the statistical significance of the two task-independent networks, we employed GAT (graph-analysis toolbox) (Hosseini et al. 2012). Brain networks have been shown to follow a specific topology known as small-worldness—an architecture that facilitates rapid synchronization and efficient information transfer (Bullmore and Sporns 2009). The clustering coefficient (*C*) and the path length (*L*) of the network are the metrics for the small-worldness (*C*/*L*). The clustering coefficient of a node is a measure of the number of edges between its nearest nodes and the average of clustering coefficient across nodes is a measure of network segregation. The path length of a network is the shortest path length between all pairs of nodes in the network—a measure of network integration. To evaluate the topology of the brain network, these parameters should be compared to the corresponding mean values of a random graph. For the comparison, 20 random graphs were generated using rewiring algorithms in GAT that preserves the topology of the graphs (the same number of nodes, total edges, and degree distribution). In a small-world network, *C* is significantly higher than that of random networks (the ratio *C* of the networks and *C* of random networks greater than 1) while *L* is comparable to random networks (the ratio *L* of the networks and *L* of random networks close to 1). Both of tractography and rsfMRI networks followed the small-world organization compared to random networks (Fig. S2).

To test the topological differences between networks, a non-parametric permutation test with 1000 repetitions was used (Bassett et al. 2008). In each repetition, the regional data of each participant were randomly reassigned to one of the two sets so that each randomized set had the same number of participants as the original sets. Then, an association matrix was obtained for each randomized set. The network measures were calculated for all the networks at each density. The differences in network measures between randomized groups were calculated resulting in a permutation distribution of difference under the null hypothesis. GAT generated the plots of between differences in network measures along with the quantified confidence intervals as a function of network density. The results demonstrated that our task-independent networks were significantly different from the random network in small-worldness, global efficiency, and modularity (*p* < 0.05) (Fig. S3) but was no difference between the tractography network and the rsfMRI network (*p*s > 0.9).

### Task-dependent fMRI and ROI analysis

To test the cognitive signature of the task-independent networks, we utilized three previous studies which investigating semantic cognition. The criteria to choose task-fMRI datasets were that the study (1) was published, (2) used the same fMRI parameters, (3) employed the distortion–correction that improves fMRI signal in the rostral temporal lobe and orbitofrontal cortex, and (4) were paired with a variety of different tasks all of which tapped different aspects of higher cognitive function other than semantic cognition.

In the first study (Visser et al. 2012), participants were asked to perform the word and picture versions of the Camel and Cactus task (CCT) (Bozeat et al. 2000) and the Pyramids and Palm Trees test (PPT) (Howard and Patterson 1992). Participants were required to decide which of the bottom pictures/words was more associated in meaning with the top picture/word by pressing a button with the corresponding finger. As a control task, visually scrambled version of the pictures/words from the semantic task was presented. In this task, participants were asked to indicate which bottom stimulus (inverted) matched the top item (non-inverted).

The second study probed the auditory modality, including auditory words and environment sounds (Visser and Lambon Ralph 2011). Participants were asked to judge whether the item was living or non-living. For the control task, pink or brown noise bursts were presented. Participants were required to indicate with a button press whether the sound had a high or low pitch.

The third study (Binney et al. 2010) used a visually presented synonym judgement task. Participants decided which of the bottom words (e.g., functional vs. receptive) was more associated in meaning with the top word (e.g., handy) by pressing a button with the corresponding finger. The matched control task was a number judgement task. Participants were asked to select which of the bottom numbers (e.g., 325 vs. 367) was closer to the top number (e.g., 358) in numerical value.

All studies had a semantic task and a control task. The semantic tasks activate both the semantic representation network as well as cognitive control regions. The non-semantic tasks covered different cognitive functions: visuospatial processing, numerical processing, or auditory processing, each of which recruits different regions. All tasks required subjects to select an appropriate answer between two choices, which recruits cognitive control functions. Thus, taken together, our task-fMRI studies cover cognitive control, semantic representation/memory, language, visuospatial function, numerical processing, and perception. These tasks did not cover all higher cognitive functions but we believe that they are sufficient to test and demonstrate the core purpose of the study.

To evaluate the cognitive signature of the task-independent networks, we used the same 43 ROIs used for the rsfMRI network analyses. We extracted regional activity according to the three contrasts; semantic contrast (semantic > control), rest contrast (rest > semantic), and control contrast (control > rest). Then, we grouped the ROIs according to the modules of each task-independent network and averaged the regional activity (Fig. S4). The level of network activity was statistically tested using one-sample *t*-test (two-tailed). Finally, we presented the pattern of activity as *Z*-scored value.

### Representational similarity analysis

To quantify and compare the “cognitive signature” for different datasets, we employed representational similarity analysis (RSA) (Kriegeskorte and Kievit 2013). RSA is pattern information analysis that compares representational geometries between brain regions, stimulus, conceptual and computational models, and behaviors of similarity (Kriegeskorte et al. 2008). In fMRI, we compare the patterns of brain activity evoked by a set of stimuli or experimental conditions to each other to characterize the geometry of a representation. The dissimilarity of two patterns induced by two different conditions corresponds to the distance between their points in the representational space. By measuring these distances, we can construct a matrix called the representational dissimilarity matrix (RDM), which indicates the degree to each pair of stimuli or conditions is distinguished. An RDM is a square symmetric matrix containing a cell for each pair of stimuli or experimental conditions and serves as the signatures of representations. RSA computes a second-order correlation (Spearman’s correlation) between model RDMs and activation-pattern RDMs. Model RDMs represent the similarity between stimuli as predicted by a computational model or hypothesis about the structure of the stimulus space. Activation-pattern RDMs are computed for a set of voxels using dissimilarity function (1-Pearson’s correlation across voxels).

In the current study, each cell (ROI) in an RDM represented a value of dissimilarity between patterns of activity across the three conditions derived from the fMRI studies. We constructed two model RDMs based on the tractography and rsfMRI networks. In the model RDMs, the pattern of activity across ROIs within a module was similar (dissimilarity ‘0’), whereas that of ROIs from different modules was dissimilar (dissimilarity ‘1’). RSA allows us to compare similarity of activation patterns to similarity pattern predicted by a theoretical model. We examined the similarity between the activation patterns of individual RDMs from three fMRI studies and the theoretical model from the task-independent networks. We also directly compared the similarity between rsfMRI RDM and tractography RDM.

## Results

### Task-independent networks results

**Fig. 2:**
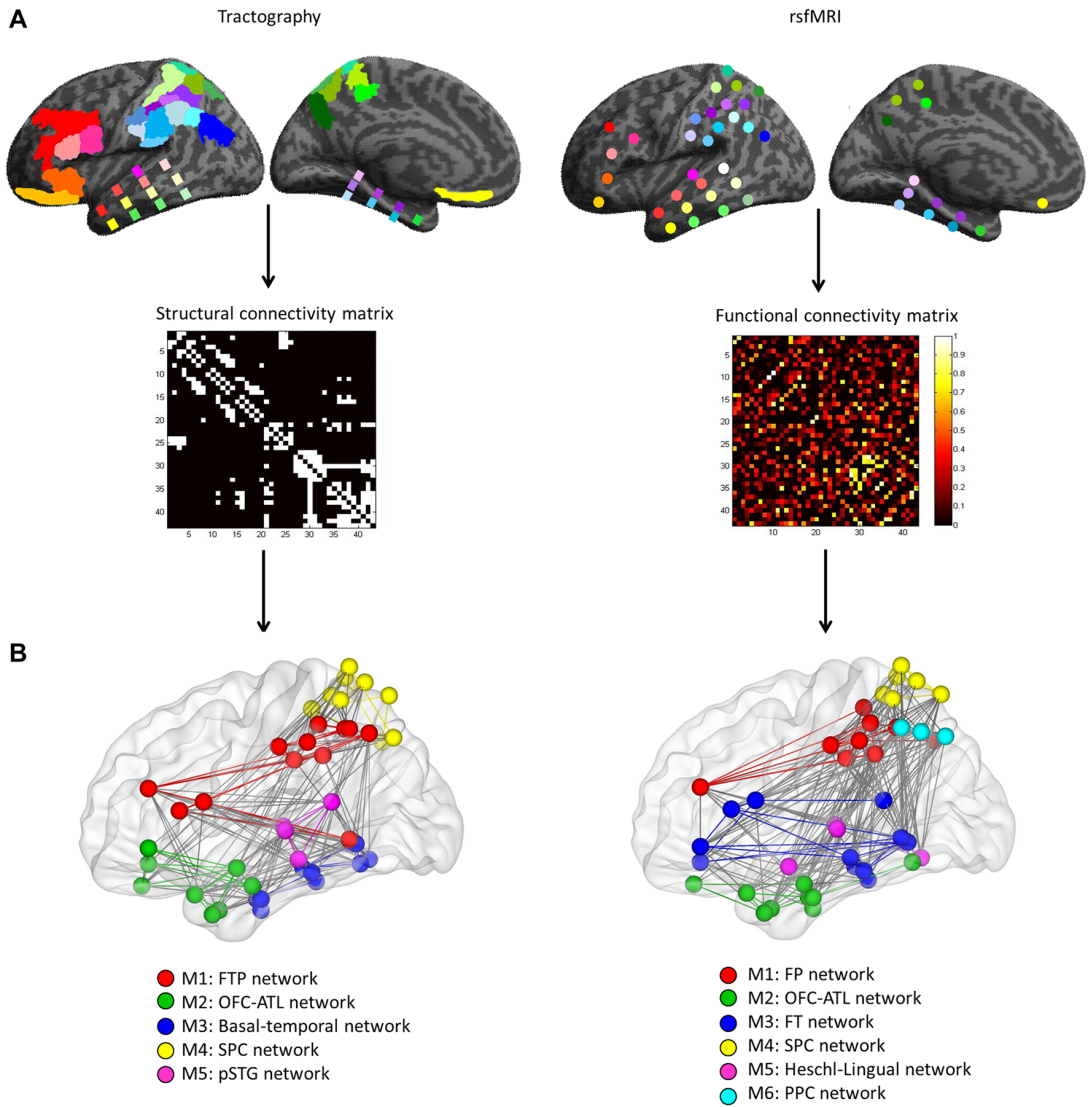
Procedure and definition of task-independent networks on the lateral associative cortices. **a** The nodes (ROIs) and edges (connections) were computed in each dataset and used to construct associative matrices. **b** Networks defined by structural connectivity (tractography) and functional connectivity (rsfMRI). Colors in nodes (ROIs) and edges (connectivity) corresponded to each networks with a unique color

To characterize the task-independent networks, distortion-corrected DWI data [a detailed description of the tractography data has been previously published (Jung et al. 2016)] and dual-echo rsfMRI data were used to define network formation within the lateral associative cortex (43 ROIs spanning frontal, lateral parietal, and temporal regions: Fig. S1; see Supplementary Information for further details). For the DWI data, probabilistic tractography was performed and the resultant connections between ROIs were used to construct a structural connectivity matrix (a binary matrix: 0—no connection; 1—connection; Fig. 2a, left). For the rsfMRI data, correlation analyses were conducted to estimate functional connectivity between ROIs and the averaged connections across the participants were used to form a functional connectivity matrix (Fig. 2a, right). The pattern of connectivity for each dataset was then examined using graph-theory analysis. To disclose modular structure within the associative cortices, we assessed a global network property—modularity. A network can be divided into modules that classify nodes (ROIs) with similar functions by disentangling the structure of the network (Bullmore and Sporns 2009). The results revealed several modules (networks) and hubs within the associative cortex in each data set (Fig. 2b; Tables S1, S2). Graph-theory analysis revealed six networks in the rsfMRI data (Fig. 2b; Table S1). A frontal-parietal (FP) network (red) was composed of DLPFC, 2 regions in SPC (5 Ci and 7M), most of IPC and IPS. An orbitofrontal–anterior–temporal lobe (OFC–ATL) network (green) consisted of medial OFC, temporal polar/anterior regions, and pFG. A frontal–temporal (FT) network (blue) was comprised of ventolateral frontal regions (BA 44, 45, 47, and latOFC) and posterior temporal lobe. A superior parietal cortex (SPC) network (yellow) covered most of the SPC regions. A Heschl-lingual network (pink) was composed of Heschl’s gyrus, STG, and lingual gyri. Finally, a posterior parietal cortex (PPC) network (cyan) only contained angular gyrus (PFcm, PGa, and PGp). Similar to the rsfMRI network results, there were five networks in the DWI data (Fig. 2b; Table S1). A frontal–temporal–parietal (FTP) network (red) was composed of lateral frontal regions (DLPFC, BA44, and BA45), IPC, IPS, and pMTG in the temporal lobe. OFC–ATL network (green) contained OFC and BA 47 in the frontal lobe and temporal polar/anterior regions. A basal–temporal network (blue) consisted of mainly ventral temporal areas, whereas an SPC network (yellow) was composed of all SPC regions and PGp. A posterior–superior temporal gyrus (pSTG) network (pink) was comprised of Heschl’s gyrus and STG.

We observed significant similarities in the formation of networks between the rsfMRI and the tractography datasets (Fig. 2b). First, the FP network (rsfMRI) and the FTP network (tractography) were highly overlapping with each other. DLPFC and IPS/IPC found in both networks are key regions in the cognitive control network (Duncan and Owen 2000; D’Esposito 2007; Spreng et al. 2013; Seeley et al. 2007). Second, the OFC–ATL network was also found in both rsfMRI and tractography data. These areas (OFC and ATL) have been implicated in semantic cognition (Binney et al. 2012; Devlin et al. 2003; Lambon Ralph et al. 2017) and various aspects of social cognition (Olson et al. 2013; Zahn et al. 2007; Bechara et al. 2000; Binney et al. 2016). Third, the SPC network was detected in both data sets. SPC has been associated with a critical role in visuomotor control including multimodal encoding of location, reaching, and grasping (for a review, see Culham et al. 2006; Goldenberg and Spatt 2009). Finally, the graph-theory analysis clustered sensory-related regions as a sub-network from the rest of the associative cortex. In the rsfMRI, a Heschl-lingual network was found and pSTG network in the tractography.

There were only a small number of examples of different networks from the tractography and rsfMRI results. The pattern of functional connectivity drew out a FT network comprising the ventrolateral prefrontal cortex (OFC, BA 44, 45, and 47) and middle/posterior temporal lobe (pSTG, MTG, ITG, and FG). These regions have been regarded as key parts of the language system, especially the ventral stream (Hickok and Poeppel 2004; Parker et al. 2005; Saur et al. 2008). Second, the pattern of structural connectivity extracted a basal–temporal network, traditionally associated with the visual “what pathway” (Goodale and Milner 1992). Finally, the clearest difference was the PPC network found in rsfMRI data only. Despite this small handful of divergent examples, when taking the entire datasets as a whole, there was no significant difference on the formation of networks between rsfMRI and tractography results (global network properties, *p*s > 0.9)—underlining, in formal terms, the strong similarity between the two task-independent networks.

### Cognitive signature of task-independent networks

**Fig. 3:**
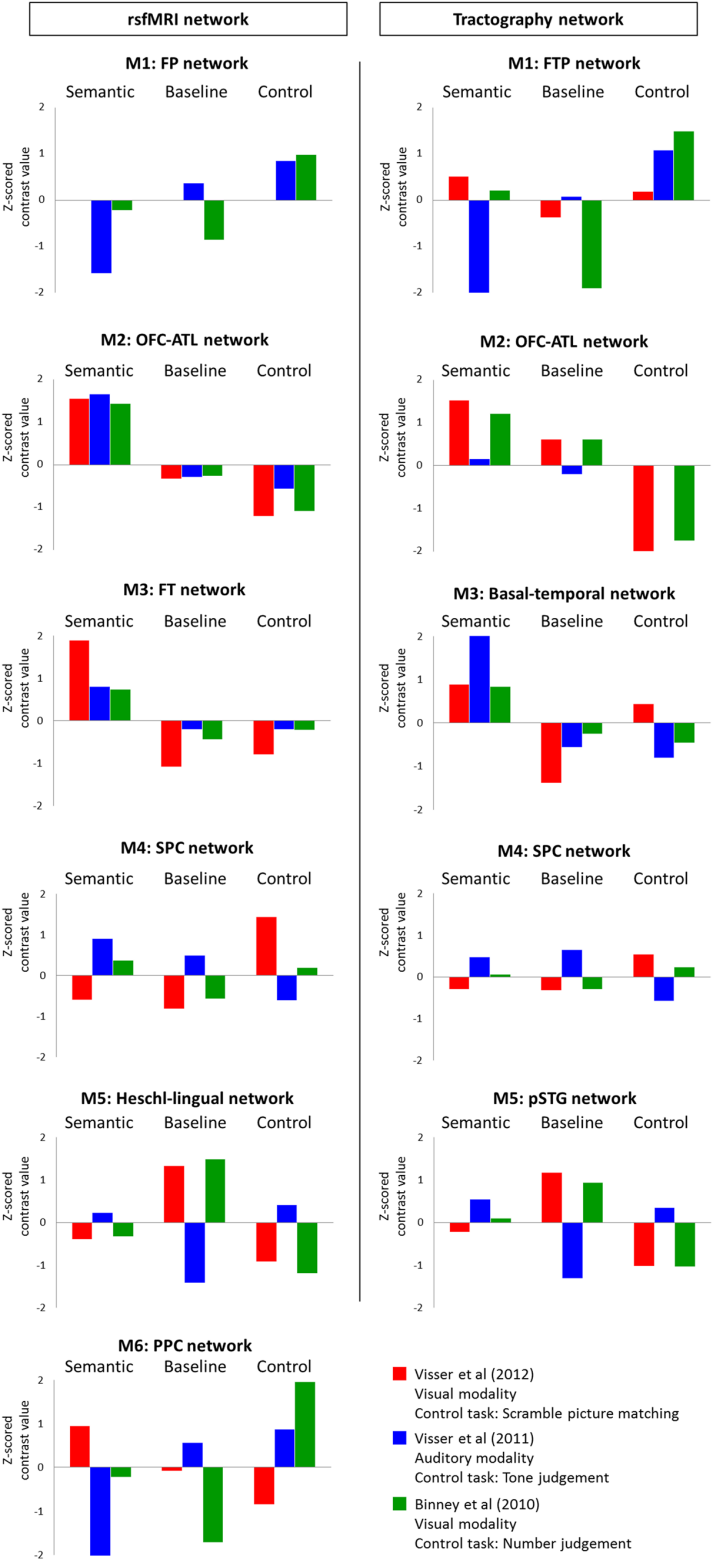
Cognitive features of networks. Left column shows rsfMRI networks and right column shows tractography networks. Bar graphs indicate *Z*-scored brain activity value according to three contrasts; semantic contrast, rest contrast, and control contrast. Each color represents three fMRI studies; red—Visser et al. (2012), blue—Visser et al. (2011) and green—Binney et al. (2010)

Based on the spatial distribution of each network, it would be possible to predict their cognitive functions. For example, the FP and FTP networks overlapped with the cognitive control network. The OFC–ATL, FT, and basal–temporal networks coincide with regions implicated in language and semantic processing, and so on. To assess the nature of the task-independent networks in a formal way, we generated their “cognitive signature” by establishing their response pattern to three contrastive task-active fMRI datasets (Binney et al. 2010; Visser et al. 2012; Visser and Lambon Ralph 2011). The three studies differed in task-modality (visual and auditory) and each dataset contained a semantic task, a rest condition (baseline) and a control task. Visser et al. (2012) employed a visually presented semantic association task [Camel and Cactus task (CCT)] (Bozeat et al. 2000) and the Pyramids and Palm Trees test (PPT) (Howard and Patterson 1992) and a scrambled picture matching as a control task. Visser et al. (2011) presented object/environment sound for a semantic judgement task (living vs. non-living) and different pitched noise for a tone judgement task as a control. Binney et al. (2010) used a synonym judgement task and a number judgement task as a control. Using the same ROIs from the task-independent networks, we extracted regional activity according to three contrasts: semantic contrast (semantic > control), rest contrast (rest > semantic), and control contrast (control > rest). The ROI activities were grouped according to the networks then averaged and transformed into *Z*-scores (Fig. 3).

The results revealed that each network had a distinctive pattern of activity dependent on the task contrast and modality. Also, the spatially overlapping networks identified in the rsfMRI and tractography data showed highly similar task-active characteristics. The FP and the FTP networks were activated for the control contrast and showed no activation or deactivation for the other contrasts, except that the FTP network was activated for the semantic contrast when stimuli were presented visually. The OFC–ATL network showed activation for the semantic contrast, smaller activation/deactivation for the rest contrast and deactivation for the control contrast. The FT and basal–temporal networks also showed a similar pattern of activity to the OFC–ATL network. They were active for the semantic contrast whereas inactive for the other contrasts, though only the basal–temporal network showed increased activity for the control contrast in the visual modality. The SPC network was activated for the scrambled picture matching task. The networks related to sensory function (Heschl-lingual and pSTG network) demonstrated a pattern of activity dependent on task-modality. Both showed increased activity for the auditory modality during tasks and decreased activity during resting, whereas the opposite pattern of activity for the visual modality. Finally, the PPC network showed a similar pattern of activity to the FP and FTP. The network was activated by the semantic contrast only for the first study (visual modality) and at the rest contrast for the second study (auditory modality). For the control contrast, it was deactivated for the scrambled picture matching whereas activated for the tone judgement and number judgement task.

### Representative similarity analysis results

**Fig. 4:**
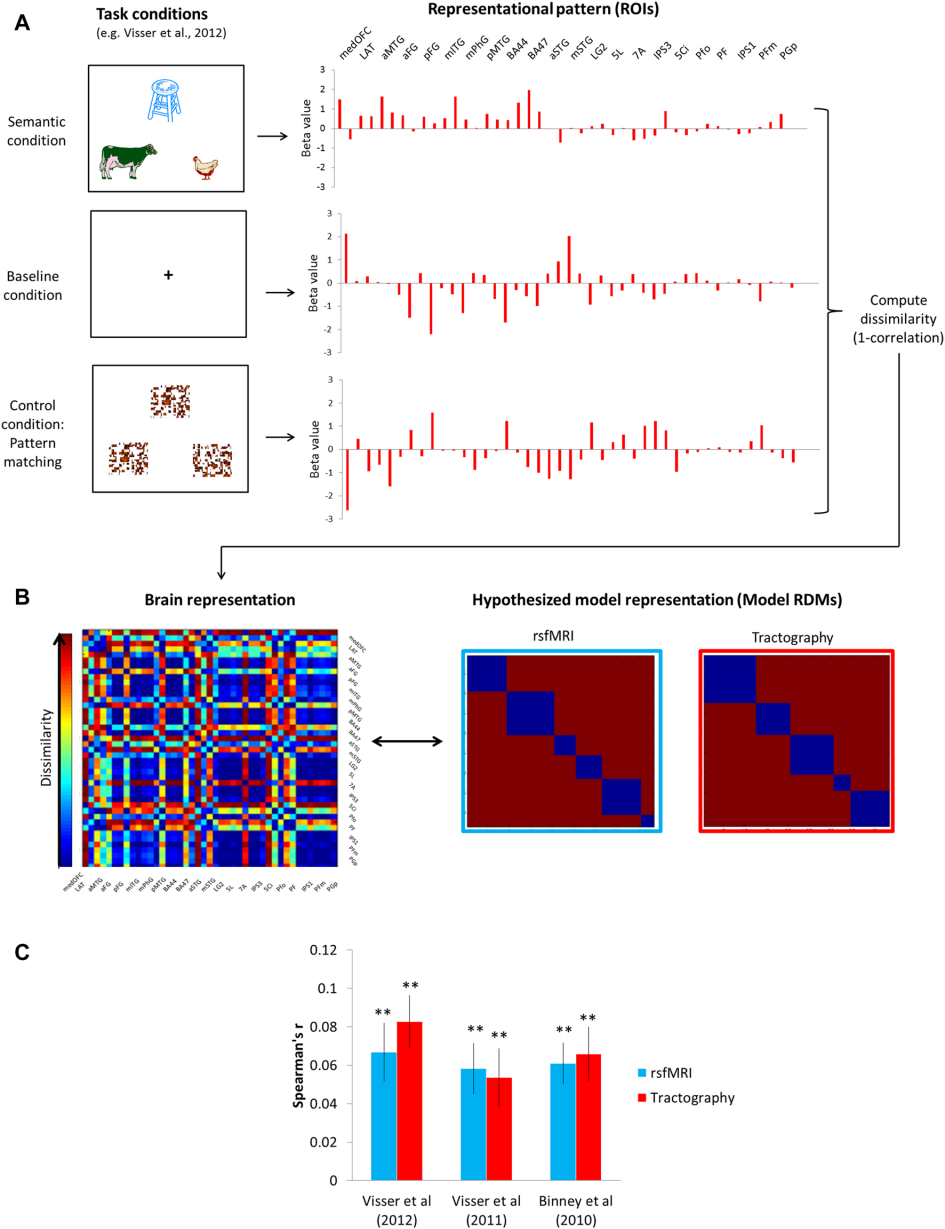
Representational similarity analysis. **a** An example of fMRI study (Visser et al. 2012). Three task conditions including semantic, control, and baseline induced activity in the ROIs (brain representations). The representation of each ROI is visualized as a set of task conditions that are active to different degrees (deactivation < 0 < activation). We computed the dissimilarity for each pair of ROIs using 1-correlation across conditions. **b** The representational dissimilarity matrix (RDM) assembles the dissimilarities for all pairs of ROIs (blue-to-red color scale). The RDM is typically symmetric about a diagonal of zeros. The RDMs were calculated for three fMRI studies at individual level. The model RDM can similarly be computed from the hypothesis for the task-independent networks. By correlating RDMs (black double arrow), we can assess to what extent the brain representation reflects experimental conditions and can be accounted for by the hypothesized model. **c** The results of RSA. The RDMs from each fMRI study were significantly correlated with the model RDMs. Light blue bars indicate the results from the rsfMRI model RDM and red bars from the tractography model RDM. **p_Bonferroni-corrected_ < 0.0001

Having formally established the contrastive cognitive fMRI signatures across the task-independent networks, we then employed representational similarity analysis (RSA) in a novel way to quantify the cognitive signature of the networks and compare the similarity of activation patterns found in task-active fMRIs to the similarity pattern predicted by task-independent networks. The three basic steps were as follows (Fig. 4): (1) to compare the brain activity patterns at the network-level, we used our ROIs instead of voxels and computed their activity according to task conditions (semantic, control, and baseline) (Fig. 4a). (2) The RDMs were constructed by computing the patterns of dissimilarity (1-Pearson’s correlation) between ROIs across the three task conditions (Fig. 4a, right). The representation in an individual is characterized by the matrix of dissimilarities between the ROIs’ representations (Fig. 4b, left). The task-independent networks were used to construct the hypothesized model RDMs (rsfMRI RDM and tractography RDM) by assuming that each network had unique pattern of activity so there would be no similarity between the networks, whereas within the networks, all nodes (ROIs) have the same pattern of activity (Fig. 4b, right). (3) Finally, we compared RDMs between the model RDMs and individual RDMs as well as between the rsfMRI and tractography RDMs.

The model RDMs showed significant correlations with the individual RDMs for all three fMRI studies (Fig. 4c); Visser et al (2012, rsfMRI: *r* = 0.067, *p* < 0.0001; tractography: *r* = 0.082, *p* < 0.0001); Visser et al. (2011, rsfMRI: *r* = 0.058, *p* < 0.0001; tractography: *r* = 0.054, *p* < 0.0001); Binney et al (2010, rsfMRI: *r* = 0.061, *p* < 0.0001; tractography: *r* = 0.066, *p* < 0.0001). Furthermore, two model RDMs showed a significant correlation (*r* = 0.264, *p* = 1.4 × 10^−10^).

**Fig. 5:**
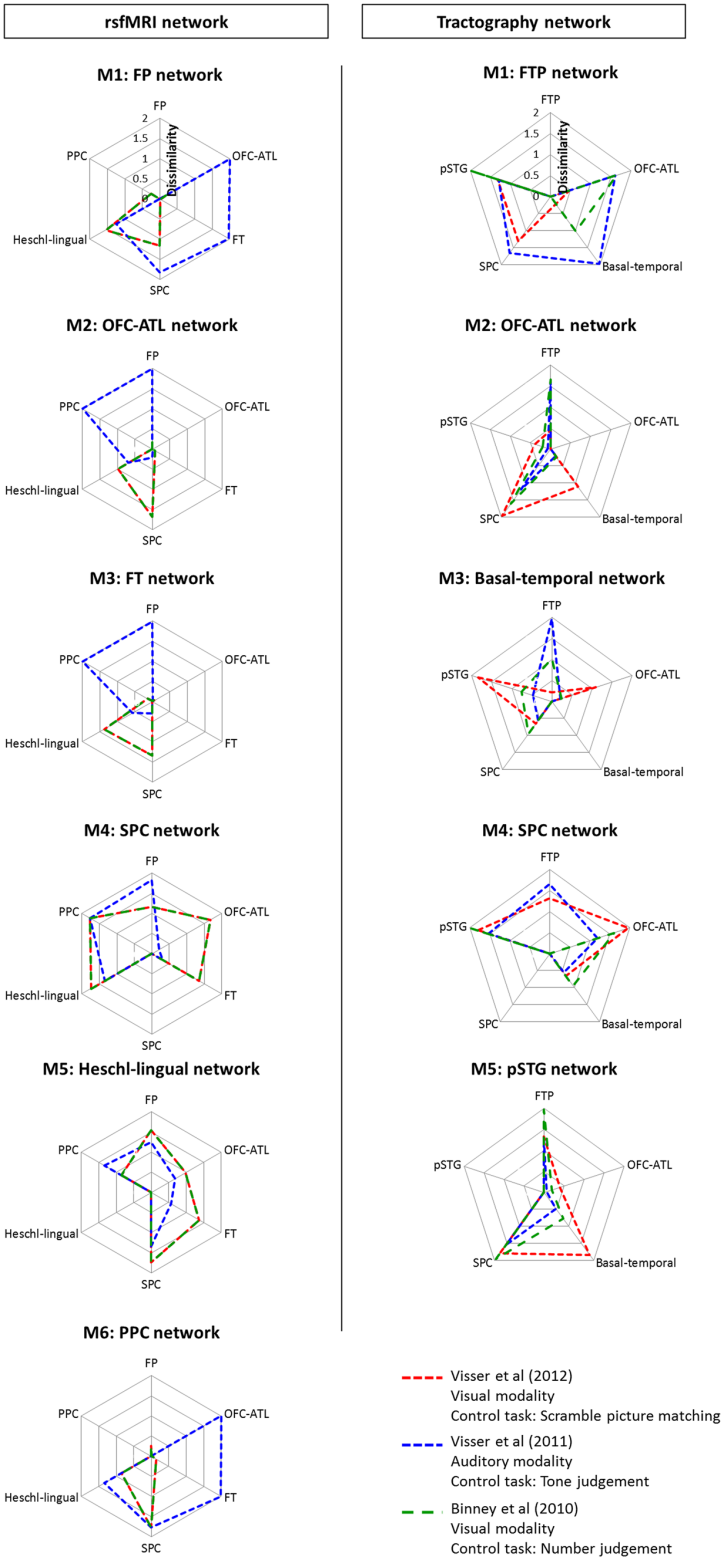
The pattern of dissimilarities of networks. Left column shows rsfMRI networks and right column shows tractography networks. Each color line represents 3 fMRI studies; red—Visser et al. (2012), blue—Visser et al. (2011) and green—Binney et al. (2010)

Figure 5 visualizes the cognitive ‘fingerprints’ across the different networks. In the rsfMRI networks, the FP network was similar to the PPC network and the OFC–ATL and FT network with visual modality only. The OFC–ATL and FT networks showed a similar pattern of dissimilarity. For the visual modality, they showed high dissimilarity to the Heschl-lingual and SPC network, whereas, for the auditory modality, to the FP and PPC network. The SPC and Heschl-lingual network were completely distinctive from the other networks but themselves. The PPC exhibited the highly similar pattern to the FP network. In the tractography networks, each network presented their own characteristics in the pattern of dissimilarity. The FTP network was different from the other networks but showed the task-dependent pattern of dissimilarity. The OFC–ATL network was similar to the basal–temporal network and the pSTG network. The basal–temporal network showed the greatest dissimilarity to the pSTG network. The SPC network was entirely different from the other networks.

## Discussion

Many studies have identified various brain networks using task-free datasets such as rsfMRI and diffusion imaging (Biswal et al. 1995; Damoiseaux et al. 2006; Beckmann et al. 2005; Fox and Raichle 2007; van den Heuvel et al. 2009; Hagmann et al. 2007; Honey et al. 2009; Jung et al. 2016). Although these studies assert functional characteristics for the identified networks, the true cognitive functions of them are rarely probed directly. This is a crucial step if we are to elucidate the relationship between distributed brain networks and higher cognitive functions. Here, we applied a new method to derive the cognitive profiles of brain networks estimated from task-free datasets. Utilizing graph-theory network analysis in two task-independent datasets (rsfMRI and DWI), we revealed the distributed connectivity networks present across frontal, parietal, and temporal associative cortices. The different functional signature of each network was then derived using three task-active fMRI datasets. Finally, we used RSA to quantify the similarity of the cognitive signature for each network across the three types of data (task-independent rsfMRI, DWI and the three task-active fMRI studies). Our results demonstrated that there was a strong association between the connectivity-based networks identified in the task-independent datasets and the pattern of network activity in the task-active fMRI datasets. Thus, our findings suggest that the topology of structural and functional connectivity in the associative cortices reflects higher cognitive functions including cognitive control, semantic representation, memory, visuospatial function, numerical processing, and perception.

We employed RSA as a new method to directly compare the task-free and task-related networks. RSA characterizes the representation in brain regions to compare the brain activity patterns representing experimental conditions to each other in fMRI (Kriegeskorte et al. 2008). It has become a popular method in brain information processing, for example, by revealing voxels corresponding to experimental conditions such as low-visual features (lines, colors) and higher visual features (faces, objects) and comparing the representations from different sources (neural activities, behaviors, and theoretical models) (Mur et al. 2009; Tyler et al. 2013; Devereux et al. 2013). We applied this method to the network-level of brain activity and successfully measured network representations (RDMs). The quantified signature of network representations for each dataset was statistically compared and demonstrated a direct relationship between task-free and task-active networks as well as between two task-free networks acquired by different neuroimaging techniques (DWI and rsfMRI). Furthermore, the RDMs captured task-related functional distinctiveness between the networks in task-active fMRI (e.g., OFC–ATL networks behave similar to the FP/FTP networks, whereas Heschl-lingual/pSTG networks are very different from other higher cognitive networks) (Fig. 5). It revealed that not only various associative cortical regions but also multiple networks are involved in higher cognitive functions (e.g., a semantic association task recruited the FP/FTP, OFC–ATL, and FT/basal–temporal networks). Thus, our results indicate that the cognitive signature of networks can be directly evaluated by utilizing a new method—RSA.

The topology of structural and functional network was not significantly different but, of course, there were some variations. There are reasons for expecting the results not to be perfectly identical—specifically the quality and nature of the two datasets are different. Sources of potential variation include: (a) even two identical sources of data measured at different times or in different ways would be expected to have different measurement noise and thus could derive non-identical clustering; (b) fMRI and DTI have fundamentally different scales of measurement of inter-node connection (continuous values between 0 and 1 vs. binary, respectively); and (c) one would expect networks during mental activity to be somewhat modulated away from the intrinsic, baseline connections. Indeed, the slight variations in clustering of the fMRI data reflect two well-known and replicated functional networks (the FT language network and the PCC).

As well as comparing the similarity of task-active fMRI, rsfMRI, and structural connectivity across the identified networks, we were also able to establish the cognitive signatures of each network—which reflected effects of task and stimulus-modality.

The OFC–ATL network was found to be specific to semantic processing: it showed activation for the semantic contrast and no activation or deactivation for the other contrasts across all three studies. The FT network derived from rsfMRI and the basal–temporal network arising in the tractography data exhibited a similar pattern of activity to the OFC–ATL network. These networks are spatially overlapping with the semantic network (Binder et al. 2009; Patterson et al. 2007; Lambon Ralph et al. 2017). The basal–temporal network also overlapped with the visual “what pathway” (Goodale and Milner 1992) and thus showed activation for the scrambled picture matching task, reflecting higher visual processing.

The FP (rsfMRI)/FTP (tractography) networks showed increased activation for all three control tasks and no activation/deactivation during rest, with the FTP network active for the semantic contrast probed using visual stimuli. The control tasks used in three fMRI studies were designed to match the level of difficulty to the paired semantic tasks. As such, the control tasks were relatively demanding and thus they recruited the cognitive control network (Duncan and Owen 2000; D’Esposito 2007; Spreng et al. 2013; Seeley et al. 2007). Both identified networks share key regions such as DLPFC and IPS/IPC with the cognitive control network and semantic control network (Noonan et al. 2013; Whitney et al. 2012; Lambon Ralph et al. 2017).

The PPC network exhibited a similar pattern of task-related activity to the FP/FTP networks. It included the posterior parietal regions (PFm and angular gyrus; AG) and partially overlapped with the semantic control network (Noonan et al. 2013). As AG is also involved in numerical and auditory processing (Seghier 2013; Humphreys and Lambon Ralph 2014), the network showed activations in the control tasks including the tone judgement task and number judgement task. The PPC network was captured only in rsfMRI dataset. It might be attributed that the AG is a component of DMN showing deactivation in certain goal-oriented tasks (Humphreys and Lambon Ralph 2014; Fox and Raichle 2007). The functional connectivity of AG can grasp its functional characteristics in response to various tasks and clustered as an independent network.

The SPC is a critical region in visuomotor control (Culham et al. 2006). In our task-related fMRI studies, only the scrambled picture matching task (Visser et al. 2012) was associated with the visuospatial processing. As we expected, the SPC network showed a strong preference for the control task from Visser et al. (2012). The Heschl-lingual and pSTG networks were sensory-related networks (Upadhyay et al. 2008). These networks showed activation during tasks and deactivation during resting for the auditory modality, and they showed the opposite pattern of activity for the visual modality.

To test and demonstrate the RSA approach, we selected three existing task-fMRI datasets for multiple reasons (see “Materials and methods”). Specifically, we considered it important (for the tractography and fMRI) to probe all parts of the cortex. Standard fMRI suffers from significant signal dropout and distortion in certain crucial regions including ventral frontal and anterior temporal areas. The data used here were collected to reduce these problems and achieve a much better coverage (Halai et al. 2014). As a result, we selected the studies published in our group because most open source fMRI data contain signal dropout and distortion. Although these studies covered cognitive control, semantic representation/memory, language, visuospatial function, numerical processing, and perception, there are other higher cognitive functions need to be probed. Future studies could employ an even wider collection of tasks to broaden the range of higher cognitive functions. These, though, would need to be collected with distortion-corrected or distortion-minimizing fMRI methods.

It should be note that our approach is based on a hard parcellation of the brain’s networks. We applied a form of hard parcellation to identity a series of subnetworks in the structural and functional data. The use of hard parcellation has a long-standing tradition in neuroscience such as using anatomical atlases (e.g., Brodmann’s cytoarchitecture maps or Automated Anatomical Labeling) or techniques parcellating brain regions (e.g., *k*-means clustering). Although these methods are widely used in the literature, other approaches are possible—such as methods which try to allow for softer boundaries or even no boundaries at all (e.g., continuous dimensions). For example, independent component analysis (ICA) is one of the most commonly used soft parcellation method allowing some overlap between independent spatiotemporal brain networks (van den Heuvel and Pol 2010). This approach extracts time and task-dependent brain networks by capturing the functional heterogeneity of a brain region. Thus, future studies can explore brain network function using these alternative approaches without constraining the boundaries of nodes/ROIs.

## Electronic supplementary material

Below is the link to the electronic supplementary material.

Supplementary material 1 (DOCX 1522 KB)
