# Supplementary material for: Establishing the cognitive signature of human brain networks derived from structural and functional connectivity
Source: Brain Struct Funct. 2018 Aug 17;223(9):4023–38. doi: 10.1007/s00429-018-1734-x (PMC6267264; doi:10.1007/s00429-018-1734-x)
Supplement: Supplementary file 1 — Supplementary material 1 (DOCX 1522 KB) [file 429_2018_1734_MOESM1_ESM.docx]

Supplementary information

**Supplementary Methods**

**1. Definition of ROIs**

The prefrontal lobe ROIs included medial orbitofrontal cortex, lateral orbitofrontal cortex, BA 44 (pars opercularis), BA 45 (pars triangularis), BA 47 (pars orbitalis), BA 46 and BA 9 (together forming dorsolateral prefrontal cortex). Probabilistic cytoarchitectonic maps from the SPM Anatomy toolbox ([Eickhoff et al., 2005](#_ENREF_20)) were used as masks for BA 44 and BA 45 to ensure that the ROIs included predominantly the cortices and a small amount of underlying gyral white matter. The orbitofrontal ROIs were defined using AAL atlas masks ([Tzourio-Mazoyer et al., 2002](#_ENREF_59)). The other frontal ROIs were defined using Brodmann grey matter masks provided by the Wake Forest University Pickatlas toolbox ([Maldjian et al., 2003](#_ENREF_38)). The parietal ROIs covered superior parietal cortex (SPC), intraparietal sulcus (IPS), and inferior parietal cortex (IPC). Probabilistic cytoarchitectonic maps from the SPM Anatomy toolbox ([Eickhoff et al., 2005](#_ENREF_20)) were used. There were seven ROIs in SPC (5L, 5M, 5Ci, 7A, 7PC, 7M, 7P), three in IPS (IPS1, IPS2, IPS3), and seven in IPC (PFop, PFt, PF, PFm, PFcm, PGa, PGp). All ROIs were resized according to their own probability in order to avoid overlapping between them. The temporal lobe ROIs were drawn on each individual’s T1 weighted anatomical imaging using MRIcro because there were no available cytoarchitectonic maps for the temporal lobe (only the large Brodmann areas without rostral-caudal distinctions). Twenty temporal lobe regions covered a polar, anterior, middle and posterior cross-section of the left temporal lobe. The temporal polar cross-section was defined by selecting the coronal slice 10 mm back from the anterior tip of the left temporal lobe and contained three ROIs, the white matter of ventromedial gyrus, the lateral gyrus and the superior (or dorsal medial) gyrus of temporal pole. The middle temporal cross-section was defined as the coronal slice at which the inferior aspect of the superior cerebellar peduncle meets the posterior wall of the pons. The position that fell halfway between these slices was defined as the anterior cross-section and was invariably at the position of the basal artery. The anterior temporal and middle temporal cross sections included the white matter of the superior temporal gyrus, the middle temporal gyrus, the inferior temporal gyrus, the fusiform gyrus and parahippocampal gyrus. The middle temporal cross-section also included the white matter of Heschl’s gyrus. The participant-specific measurement (half of the distance between the middle and polar section) was used to define the location of the posterior temporal cross-section by applying it posteriorly to the middle cross-section and included the white matter of the superior temporal gyrus, the middle temporal gyrus, the inferior temporal gyrus, the fusiform gyrus and a lateral lingual gyrus and a more medial lingual gyrus.

**Supplementary Figure 1**

**
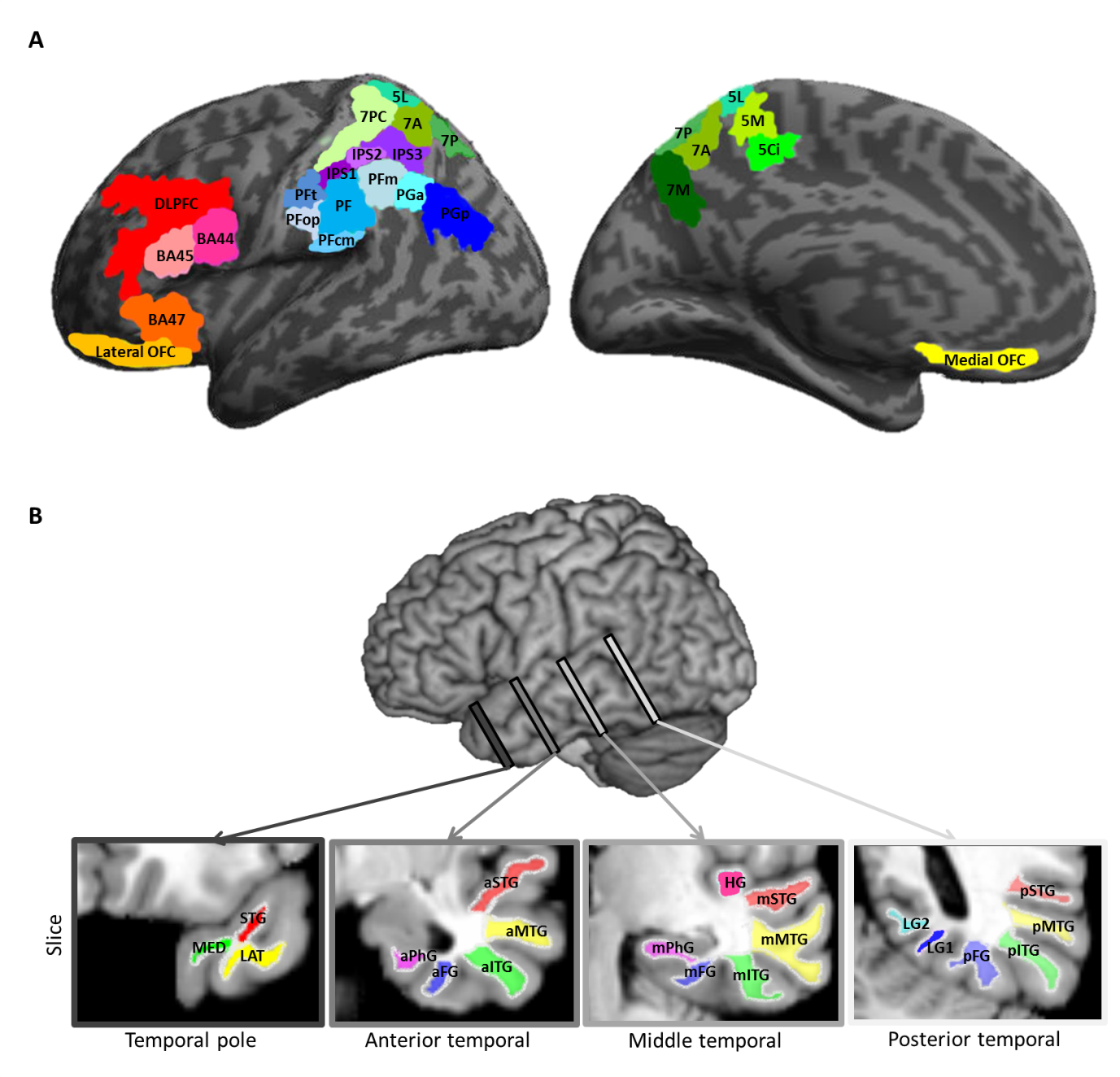
**

**Figure S1** Regions of interest (ROIs). (A) 23 frontal and parietal lobe ROIs. (B) 20 temporal lobe ROIs. STG = superior temporal gyrus; LAT = lateral temporal pole; MED = medial temporal pole; MTG = middle temporal gyrus; ITG = inferior temporal gyrus; FG = fusiform gyrus; PhG = parahippocampal gyrus; HG = Heschl’s gyrus; LG1 = lingual gyrus next to fusiform gyrus; LG2 = medial lingual gyrus; DLPFC = dorsolateral prefrontal cortex; BA = Brodmann’s areas; OFC =orbitofrontal cortex; p.Op = pars opercularis; p.Tri = pars triangularis; p.Orb = pars orbitalis; IPS =intraparietal sulcus; 5Ci, 5M, 5L = BA 5 (superior parietal cortex); 7PC, 7A, 7P, 7M = BA 7 (superior parietal cortex); PFop, PFt, PF, PFcm, PFm = supramarginal gyrus; PGa, PGp = angular gyrus; STG = superior temporal gyrus; LAT = lateral temporal pole; MED = medial temporal pole; MTG = middle temporal gyrus; ITG = inferior temporal gyrus; FG = fusiform gyrus; PhG = parahippocampal gyrus; HG = Heschl’s gyrus; LG1 = lingual gyrus next to fusiform gyrus; LG2 = medial lingual gyrus; a = anterior temporal; m = middle temporal; p = posterior temporal. This figure is adapted from Jung et al ([2016](#_ENREF_38)).

**Supplementary Figure 2**

**
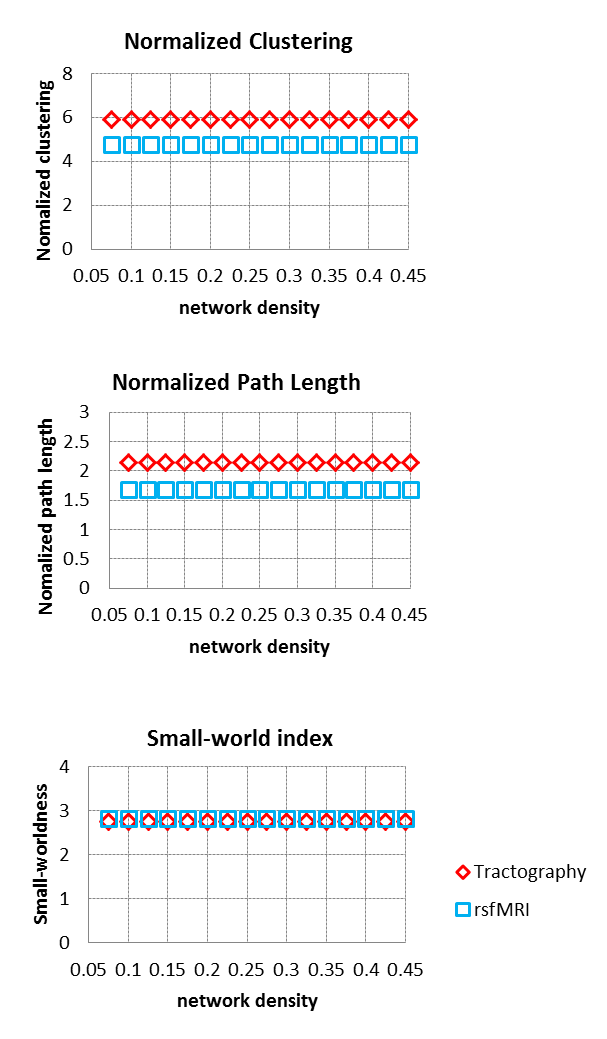
**

**Figure S2** Results of network verification. Normalized clustering, normalized path length, and small-world index of both the tractography and rsfMRI networks. Both the networks follow a small-world organization (i.e. normalized clustering of greater than 1 and normalized path length of close to 1).

**Supplementary Figure 3**

**
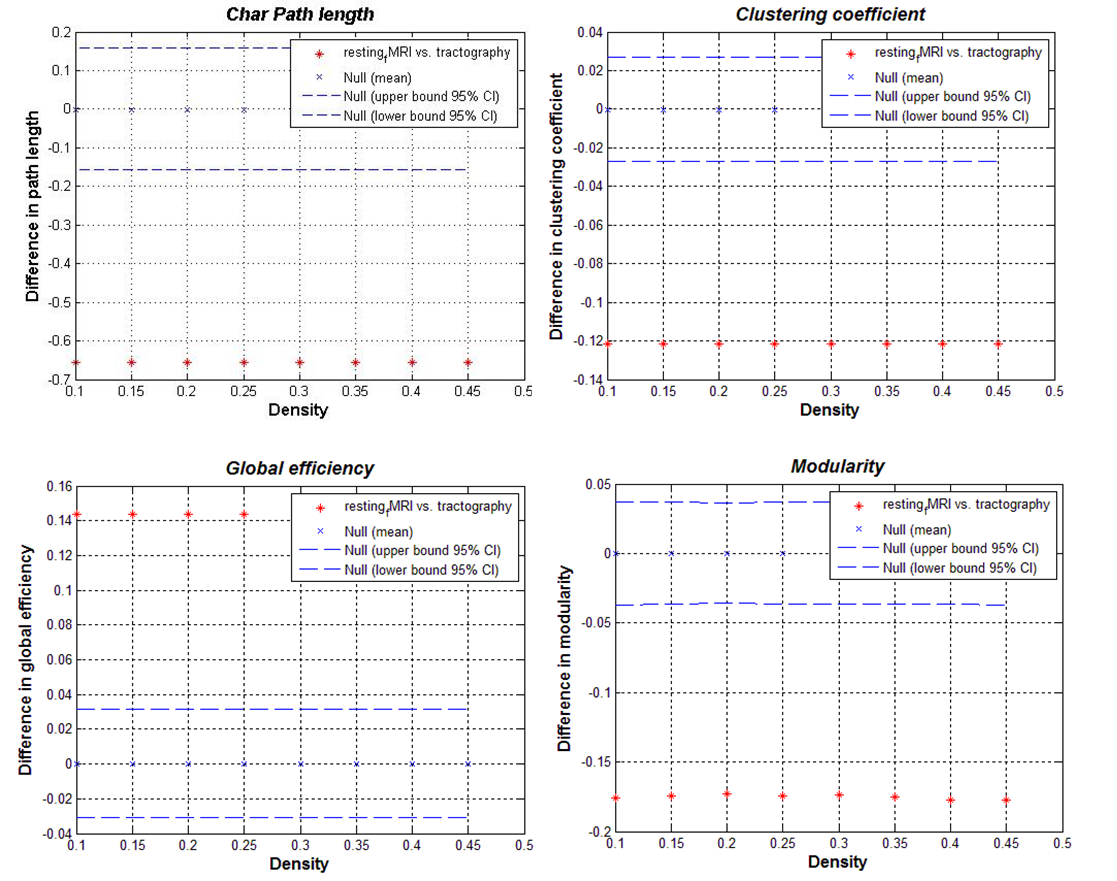
**

**Figure S3** Results of network comparison with random networks. The 95% confidence intervals and between-network differences in normalized path length (top left), normalized clustering (top right), global efficiency (bottom left) and modularity (bottom right). The red + marker shows the difference between tractography vs. rsfMRI networks; the + signs falling out of the confidence intervals indicate the densities in which the difference is significant. The positive values indicate rsfMRI > tractography and negative values indicate tractography < rsfMRI.

**
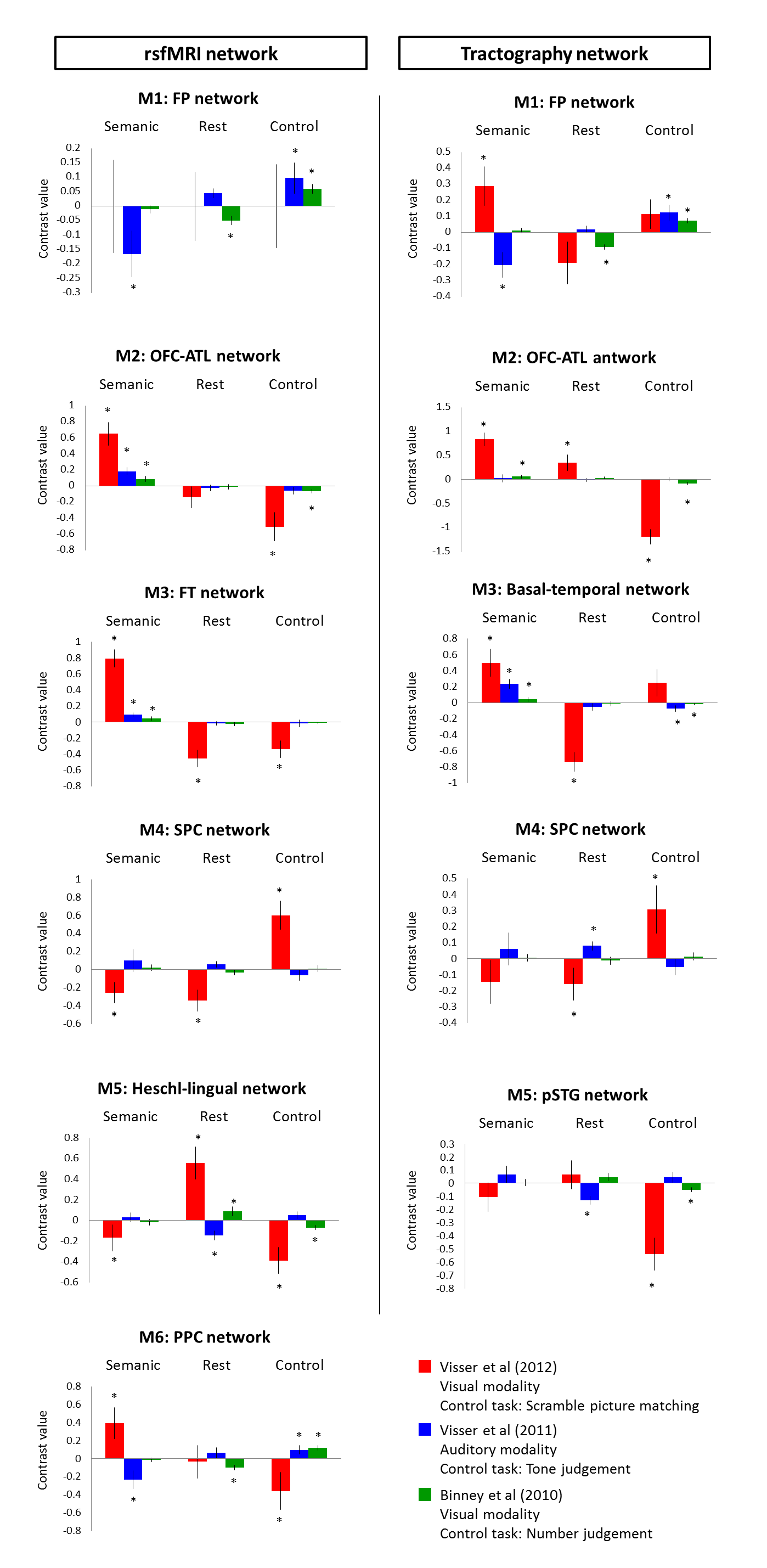
Supplementary Figure 4**

**Figure S4** The patterns of activity of networks. Left column shows rsfMRI networks and right column shows tractography networks. Bar graphs indicate brain activity value according to 3 contrasts; semantic contrast, rest contrast and control contrast. Each colour represents 3 fMRI studies; Red - Visser et al (2012), Blue - Visser et al (2011) and Green - Binney et al (2010). * p < 0.05 (one-sample t-test).

**Table S1** The summary of task-independent network analysis.

| Node | rsfMRI network | |  | Tractography network | |
| --- | --- | --- | --- | --- | --- |
|  | Modularity | Betweenness Centrality |  | Modularity | Betweenness Centrality |
| DLPFC | 1 | 3.33 |  | 1 | 9.60 |
| BA44 | 3 | 3.20 |  | 1 | 2.52 |
| BA45 | 3 | 3.06 |  | 1 | 1.36 |
| BA47 | 3 | 3.26 |  | 2 | 6.83 |
| latOFC | 3 | 3.22 |  | 2 | 1.95 |
| medOFC | 2 | 2.77 |  | 2 | 1.92 |
| 5Ci | 1 | 2.73 |  | 4 | 0.14 |
| 5M | 4 | 2.54 |  | 4 | 0.20 |
| 5L | 4 | 2.73 |  | 4 | 0.75 |
| 7PC | 4 | 2.91 |  | 4 | 14.97 |
| 7A | 4 | 2.47 |  | 4 | 2.34 |
| 7P | 4 | 3.26 |  | 4 | 0.97 |
| 7M | 1 | 2.90 |  | 4 | 9.14 |
| IPS1 | 1 | 3.26 |  | 1 | 2.46 |
| IPS2 | 1 | 2.93 |  | 1 | 0.87 |
| IPS3 | 4 | 3.35 |  | 4 | 2.80 |
| PFo | 1 | 3.11 |  | 1 | 0.56 |
| PFt | 1 | 3.19 |  | 1 | 0.43 |
| PF | 1 | 3.23 |  | 1 | 3.72 |
| PFm | 6 | 3.13 |  | 1 | 0.60 |
| PFcm | 1 | 3.07 |  | 1 | 4.74 |
| PGa | 6 | 3.07 |  | 1 | 1.03 |
| PGp | 6 | 3.22 |  | 4 | 1.24 |
| STG | 2 | 3.08 |  | 2 | 1.29 |
| LAT | 2 | 3.22 |  | 2 | 2.34 |
| MED | 2 | 2.98 |  | 2 | 2.71 |
| aSTG | 5 | 2.85 |  | 2 | 2.25 |
| aMTG | 2 | 3.38 |  | 2 | 2.43 |
| aITG | 2 | 3.09 |  | 3 | 3.95 |
| aFG | 2 | 3.02 |  | 3 | 1.55 |
| aPhG | 2 | 2.67 |  | 3 | 1.83 |
| HG | 5 | 2.79 |  | 5 | 0.89 |
| mSTG | 5 | 2.68 |  | 5 | 1.55 |
| mMTG | 3 | 3.19 |  | 5 | 7.42 |
| mITG | 3 | 2.73 |  | 3 | 3.99 |
| mFG | 3 | 3.16 |  | 3 | 2.07 |
| mPhG | 3 | 2.55 |  | 3 | 4.85 |
| pSTG | 3 | 2.84 |  | 5 | 1.51 |
| pMTG | 3 | 2.92 |  | 1 | 4.58 |
| pITG | 3 | 3.30 |  | 3 | 6.18 |
| pFG | 2 | 2.88 |  | 3 | 3.39 |
| LG1 | 5 | 2.83 |  | 3 | 1.84 |
| LG2 | 5 | 2.94 |  | 3 | 1.23 |

DLPFC = dorsolateral prefrontal cortex; BA = Brodmann’s areas; medOFC = medial orbitofrontal cortex; latOFC= lateral orbitofrontal cortex; p.Op = pars opercularis; p.Tri = pars triangularis; p.Orb = pars orbitalis; IPS =intraparietal sulcus; 5Ci, 5M, 5L = BA 5 (superior parietal cortex); 7PC, 7A, 7P, 7M = BA 7 (superior parietal cortex); PFop, PFt, PF, PFcm, PFm = supramarginal gyrus; PGa, PGp = angular gyrus; STG = superior temporal gyrus; LAT = lateral temporal pole; MED = medial temporal pole; MTG = middle temporal gyrus; ITG = inferior temporal gyrus; FG = fusiform gyrus; PhG = parahippocampal gyrus; HG = Heschl’s gyrus; LG1 = lingual gyrus next to fusiform gyrus; LG2 = medial lingual gyrus; a = anterior temporal; m = middle temporal; p = posterior temporal

**Table S2** Hubs found in task-independent network analysis

| rsfMRI network |  | Tractography network |
| --- | --- | --- |
| aMTG |  | mMTG |
| **pITG** |  | **pITG** |
| **DLPFC** |  | **DLPFC** |
| **BA47** |  | **BA47** |
| 7P |  | 7PC |
| IPS1 |  | 7M |
| IPS3 |  |  |

Hubs were defined as a node 1SD higher than the mean betweenness centrality (normalized).

Bold font represents overlapping hubs between networks.
